# *KRAS* mutations: variable incidences in a Brazilian cohort of 8,234 metastatic colorectal cancer patients

**DOI:** 10.1186/1471-230X-14-73

**Published:** 2014-04-10

**Authors:** Carlos Gil Ferreira, Veronica Aran, Ilana Zalcberg-Renault, Ana Paula Victorino, Jonas H Salem, Martin H Bonamino, Fernando M Vieira, Mariano Zalis

**Affiliations:** 1 Progenética Diagnósticos Moleculares, Av. Presidente Vargas, 962 3 Andar, Cep: 20071-002 Rio de Janeiro, RJ, Brazil; 2 Brazilian National Cancer Institute (INCA); Coordenação de Pesquisa Clínica e Incorporação Tecnológica, Rio de Janeiro, Brazil; 3 Divisão de Medicina Experimental, Brazilian National Cancer Institute (INCA), Rio de Janeiro, Brazil; 4 Instituto COI de Educação e Pesquisa, Rio de Janeiro, Brazil

**Keywords:** *KRAS*, Mutation, Gender, Cohort, Colorectal cancer

## Abstract

**Background:**

*KRAS* mutations are frequently found in colorectal cancer (CRC) indicating the importance of its genotyping in the study of the molecular mechanisms behind this disease. Although major advances have occurred over the past decade, there are still important gaps in our understanding of CRC carcinogenesis, particularly whether sex-linked factors play any role.

**Methods:**

The profile of *KRAS* mutations in the Brazilian population was analyzed by conducting direct sequencing of *KRAS* codons 12 and 13 belonging to 8,234 metastatic CRC patient samples. DNA was extracted from paraffin-embedded tissue, exon 1 was amplified by PCR and submitted to direct sequencing. The data obtained was analysed comparing different geographical regions, gender and age.

**Results:**

The median age was 59 years and the overall percentage of wild-type and mutated *KRAS* was 62.8% and 31.9%, respectively. Interestingly, different percentages of mutated *KRAS* patients were observed between male and female patients (32.5% versus 34.8%, respectively; p = 0.03). *KRAS* Gly12Asp mutation was the most prevalent for both genders and for most regions, with the exception of the North where Gly12Val was the most frequent mutation found.

**Conclusions:**

To the best of our knowledge this is one of the largest cohorts of *KRAS* genotyping in CRC patients and the largest to indicate a higher incidence of *KRAS* mutation in females compared to males in Brazil. Nevertheless, further research is required to better address the impact of gender differences in colorectal cancer.

## Background

Personalised medicine is an evolving field that seeks to target cancer therapies based on unique genetic characteristics of the tumour and/or the patient [1]. One of the most significant advances towards personalised care in the field of oncology was the establishment of *KRAS* gene mutation as a validated biomarker predicting efficacy in epidermal growth factor receptor (EGFR) targeted therapies – such as cetuximab and panitumumab - in the treatment of metastatic colorectal cancer (mCRC) [2,3]. According to Siegel et al. [4], CRC is the third cause of new cancer cases and of death by cancer in the United States with an estimate of 73,420 new cases for males and 70,040 new cases for females in 2012. In Brazil, data published by the Brazilian National Cancer Institute predicted the number of new cases for 2012 of 14,180 for males and 15,960 for females with a frequency variation depending on the country’s region (data available online at http://portal.saude.sp.gov.br/resources/ses/perfil/gestor/homepage/estimativas-de-incidencia-de-cancer-2012/estimativas_incidencia_cancer_2012.pdf). The Southeast region shows the highest incidence of CRC, being the second most frequent cause of cancer for both men (22/100,000) and women (23/100,000). The differences observed in the incidence of CRC according to different Brazilian regions could relate to the idea that differences in patients origins might contribute to the incidence of somatic mutations in candidate cancer genes such as *KRAS*[5].

In order to understand *KRAS* function it is important to address how Ras proteins are activated. The Ras protein family belongs to a group of small GTPases, which are able to cycle between an inactive (GDP-bound form) and an active state (GTP-bound form) leading to the activation of several effector kinases. These proteins are involved in cell proliferation, differentiation and survival, hence its importance in cancer research [6]. Mutations in *RAS* proto-oncogenes (comprising *H-, N-* and *K-RAS*) are among the most common in malignant tumours and although *RAS* isoforms are very similar, *KRAS* is more frequently found mutated in cancers occurring in 22% of all tumours analysed compared to 8% for *NRAS* and 3% for *HRAS*[7]. In mCRC, mutation in *KRAS* gene result in continuous activation of intracellular EGFR pathway regardless the pharmacological blocking of the receptor [8]. Thus, proliferation, invasion, survival and metastasis of the tumour are maintained. Clinically, patients with *KRAS* wild-type tumours are more likely to respond to anti-EGFR therapy whereas those with mutant *KRAS* show lack of benefit [9-14]. Therefore, anti-EGFR monoclonal antibodies are only indicated in patients with *KRAS* wild-type tumours [15].

Since June 2008, a Merck Serono Oncology sponsored program began to reimburse for *KRAS* mutation analyses in mCRC patients in Brazil. Almost all *KRAS* mutations tests were performed in a single molecular biology facility. A total of 8,234 patients had their primary tumour and/or metastasis analysed through the program. Here, some epidemiological characteristics of the *KRAS* mutations are described.

## Methods

### Ethics statement

The local ethical committee (Comitê de Ética em Pesquisas- CEP from Hospital Pró-Cardíaco, Esho Empresa de Serviços Hospitalares) was consulted and approved the analysis and publication of the epidemiological data without patient’s individual informed consent.

### Merck Serono Oncology KRAS Program in Brazil

Launched in June 2008, the *Merck Serono Oncology KRAS Program* invited physicians to request *KRAS* tests for any patient diagnosed with mCRC. Initially, the program was based in written formulary requests and telephone/fax contacts to provide authorizations for tests to be performed. In October 2009, the program was upgraded to internet-based process. All physicians had to accept the terms of the program, which comprehended no obligation in any kind of prescription and clarification to the patient that a pharmaceutical industry was covering the costs of the tests, having no contact with the identity of the patients or further individual results. After authorization was granted, the biological material (paraffin blocks and slides) was sent to the laboratory facility using Brazilian mail express services (SEDEX). The results were confidential and sent directly from the laboratory to the physician without report to Merck Serono.

### DNA extraction

The tissue slide corresponding to the paraffin-embedded tumour block was analysed by a trained pathologist. The tumour area was marked and a fragment was digged out from the tissue block using proper stylet. The DNA extraction was performed using the commercial kit MagneSil™ (Promega Corporation, Madison, Wisconsin, USA) following manufacturer instructions described elsewhere.

### Polymerase chain reaction (PCR)

Extracted DNA was analysed with semi-nested PCR. The primers utilized were: KRASF1 e KRASR. The first stage reactions were accomplished with 5 μl of DNA, 2 μl MgCl (50 mM), 5 μl Promega 10× PCR Buffer, 2 μl KRASF1 primer (5′-GTGTGACATGTTCTAATATAGTCA-3′) (50 pmol/μl), 2 μl KRASR primer (5′-GAATGGTCCTGCACCAGTAA-3′) (50 pmol/μl), 5 μl sNTPS (2,5 mM), 28,5 μl distilled water, 0,5 μl Taq Platinum DNA Pol (Invitrogen) in total volume of 50 μl, following a cycle program of 94°C for 1 min, 40 cycles of 95°C during 20 seconds, 60°C during 30 seconds and 72°C for 1 min and 30 seconds and final extension of 20 min in 72°C. For the semi-nested stage, the products from first stage PCR were diluted in 1:100 ratio in distilled water and 5 μl were added to a mixture containing 5 μl of DNA, 2 μl MgCl (50 mM), 5 μl Promega 10 × PCR Buffer, 2 μl KRASF primer (50pmol/μl), 2 μl KRASF2 primer 5′-TCATTATTTTTATTATAAGGCCTGCTG-3′ (50 pmol/μl), 5 μl sNTPS (2,5 mM), 28,5 μl distilled water, 0,5 μl Taq Platinum DNA Pol (Invitrogen) in total volume of 50 μl, in cycle conditions of 94°C during 1 min, 40 cycles in 95°C for 20 seconds, 58°C for 30 seconds and 72°C for 1 min and 30 seconds. All reactions were performed using MyCycler™ Thermal Cycler (BioRad Laboratories, Inc; Hercules, CA, USA) equipment.

The PCR products were analysed in a 2% agarose gel and visualized under ultraviolet light. The samples were considered positive when the band correspondent to 185 base pairs was seen. After agarose gel electrophoresis, 40 μl of PCR substrate was purified using commercial kit GFX™ PCR DNA and Gel Purification Kit (GE Healthcare, Piscataway, NJ, USA) following manufacture’s instructions.

### DNA sequencing

The amount of 2 μl of substrate was used in sequencing reactions using commercial kit BigDye Terminator v.3.1 Cycle Sequencing Kit (Applied Biosystems) according to manufacture’s instruction using 3.2 pmol of oligo KRASR (5′-GAATGGTCCTGCACCAGTAA-3′).

The sequences were analysed using ABI PRISM® 3100 GeneticAnalyzer/HITACHI (Applied Biosystems) and the presence of mutations were performed using the Mutation Surveyor (Softgenetics) software.

### Statistical analysis

Parameters evaluated were frequency of mutations, frequency of mutations per codons (12 or 13), types of mutations, gender, age, and geographic region distributions. Cross tabulation of interests combining some of the mentioned parameters were analysed whenever considered of interest.

Statistical analysis was performed using the Statistical Package for Social Sciences (SPSS software, version 13.0 for Windows, Chicago, IL, USA). The level of significance for p value was established as below 5%.

Continuous variables were presented using mean values and standard deviation. The categorical variables were presented as absolute frequency and percentages. Further associations between variables were verified through Pearson’s chi-square test.

## Results

### Total frequency of wild-type versus mutant *KRAS*

**Figure 1:**
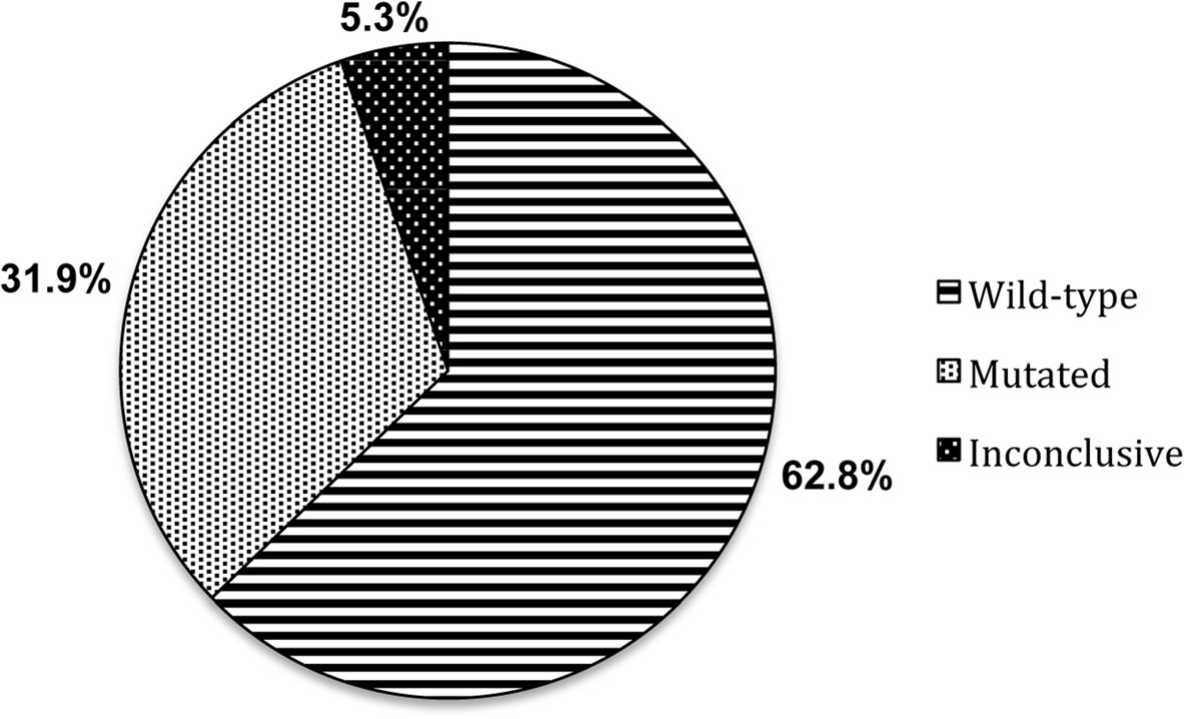
**Overall percentage of mutant versus wild-type *KRAS *cases.** A total of 8,234 samples were pooled for analysis and 437 tests presented an inconclusive *KRAS* diagnostic.

A total of 8,234 samples were pooled for analysis. There were 437 tests without a conclusive *KRAS* diagnostic. This occurred mostly due to inadequate samples that prevented DNA amplification related to fixation duration, type of formalin previously used and/or insufficient tumour availability. Whenever this occurred, up to four attempts of DNA extraction and amplification were performed before the physician requested more samples for analysis. Figure 1 displays the total frequencies of *KRAS* mutations. Our results show an overall *KRAS* mutation frequency of 31.9% (n = 8,234).

### Analysis of *KRAS* according to regions

Brazil has a territory of continental size showing a strong ancestral diversity: the result of interethnic crosses between different populations: the European colonizers (mainly Portuguese), African slaves and Amerindians [16]. For example, the Northeast region has a history of strong African presence due to slavery, the South was mostly settled by European immigrants and the North by Amerindians [17].

The Brazilian population admixture has important clinical implications [17]. Different population origins may determine different trends in gene mutations, therefore we analysed samples coming from patients from the five different regions of Brazil. The distribution of *KRAS* mutations according to geographic region is shown in Table 1. Each region showed more than 30% incidence of *KRAS* mutant cases, being the Southeast the highest and the Northeast region the lowest in incidence. The distribution between genders of study population was well balanced between female (48.1%) and male (51.9%). The mean age of the population analysed was 58.8 years (standard deviation 13.02 range 14 to 102). The mean age for the presence of mutations was 60 years and for its absence, 59 years.

**Table 1:** **Geographic distribution of *KRAS *** **status according to region**

**Geographic distribution**	**Wild-type**	**Mutant**	**Total n (%)**
	**n (%)**	**n (%)**	**8,234 (100)**
Southeast	3,026 (65.3)	1,608 (34.7)	4,634 (56.3)
South	1,091 (68)	514 (32)	1,605 (19.5)
Northeast	558 (69.1)	250 (30.9)	808 (9.8)
Middle west	387 (66.5)	195 (33.5)	582 (7.1)
North	93 (65.5)	49 (34.5)	142 (1.7)
Missing data	-	-	463 (5.6)

### Types of mutations and their geographical distribution

After considering the different geographical regions and obtaining the result of mutant versus wild-type samples per region, we performed genotyping of *KRAS* codons 12 and 13, which are the most commonly affected codons in CRC also known to be predictors of resistance to anti-EGFR therapies [3]. By doing this, we accessed the frequent aminoacid changes. Although recent studies have analysed the importance of codon 61 (less frequently found mutated in CRC) [18], at the time we started our study mutational analysis of *KRAS* codons 12 and 13 was standard for patients with mCRC.

The correlation between geographic distribution and *KRAS* mutations was available for 8,208 tests (99.7% of all performed tests). Overall, the mutation Gly12Asp (GGT > GAT) was the highest in most regions with the exception of the North where Gly12Val (GGT > GTT) was the most frequent. The Middle West region showed the highest percentage of Gly12Asp (GGT > GAT) mutations, whereas the North showed the least. At codon 13, the most prevalent modification was Gly13Asp (GGC > GAC), followed by other low frequent alterations. The percentage of mutations according to each region ranged from 30.9% (Northeast) to 34.7% (Southeast). Details on the distribution and types of mutations are described in Table 2.

**Table 2:** **Incidence of *KRAS *** **mutations according to region**

**Mutation**	**Middle west n (%)**	**Northeast n (%)**	**North n (%)**	**Southeast n (%)**	**South n (%)**
	**195 (100)**	**250 (100)**	**49 (100)**	**1,610 (100)**	**515 (100)**
Gly12Asp (GGT > GAT)	79 (40.5)	99 (39.6)	15 (30.6)	587 (36.5)	174 (33.8)
Gly12Val (GGT > GTT)	48 (24.6)	53 (21.2)	16 (32.7)	397 (24.7)	124 (24.1)
Gly12Cys (GGT > TGT)	14 (7.2)	21 (8.4)	6 (12.2)	114 (7.1)	50 (9.7)
Gly12Ala (GGT > GCT)	12 (6.2)	14 (5.6)	1 (2)	90 (5.6)	32 (62)
Gly12Ser (GGT > AGT)	12 (6.2)	24 (9.6)	4 (8.2)	125 (7.8)	29 (5.6)
Gly12Arg (GGT > CGT)	6 (3.1)	5 (2)	2 (4.1)	16 (1)	5 (1)
Gly13Asp (GGC > GAC)	24 (2.3)	34 (13.6)	4 (8.2)	264 (16.4)	96 (18.6)
Gly13Cys (GGC > TGC)	0	0	0	11 (0.7)	2 (0.4)
Gly13Ser (GGC > AGC)	0	0	1 (2)	3 (0.2)	1 (0.2)
Gly13Val (GGC > GTT)	0	0	0	1 (0.1)	1 (0.2)
Gly13Arg (GGC > CGC)	0	0	0	2 (0.1)	1 (0.2)
**Total/region (100)**	33.5%	30.9%	34.5%	34.7%	32.0%

### Distribution of different *KRAS* mutations according to gender

Gender was tested as one of the factors that could be relevant in our analysis since it is known that in different populations, women and men are affected differently by CRC, where men are at higher risk [19]. Table 3 shows that mutations in codon 12 were much more prevalent than in codon 13 (83% and 17%, respectively) and the distribution of mutations according to both codons did not differ by gender (p = 0.34). Overall, the most frequent point mutation in codon 12 was Gly12Asp (GGT > GAT), being found in 36.4% of all mutant samples followed by Gly12Val (GGT > GTT) (24.3%) and Gly12Cys (GGT > TGT) (7.9%). In codon 13, Gly13Asp (GGC > GAC) was the most common mutation (16.1%). The *KRAS* mutation profile according to gender correlated well with the overall *KRAS* mutant type, being Gly12Asp (GGT > GAT) the most frequent mutation in each gender. A detailed description of the mutations identified is shown in Table 3.

**Table 3:** **Frequency of *KRAS *** **mutations and types of alterations according to gender**

***KRAS *** **mutation type**	**Female n (%)**	**Male n (%)**	**Total mutation n (%)**
	**1,305 (100)**	**1,318 (100)**	**2,623 (100)**
**Codon 12**	1,082 (83)	1,093 (83)	2,175 (83)
Gly12Asp (GGT > GAT)	457 (35)	498 (37.8)	955 (36.4)
Gly12Val (GGT > GTT)	312 (23.9)	325 (24.7)	637 (24.3)
Gly12Ser (GGT > AGT)	102 (7.8)	92 (7)	194 (7.4)
Gly12Ala (GGT > GCT)	78 (6)	71 (5.4)	149 (57)
Gly12Cys (GGT > TGT)	112 (8.6)	94 (7.1)	206 (7.9)
Gly12Arg (GGT > CGT)	21 (1.6)	13 (1)	34 (1.3)
**Codon 13**	223 (17)	224 (17)	447 (17)
Gly13Asp (GGC > GAC)	215 (16.5)	208 (15.8)	423 (16.1)
Gly13Cys (GGC > TGC)	3 (0.2)	10 (0.8)	13 (0.5)
Gly13Ser (GGC > AGC)	3 (0.2)	3 (0.2)	6 (0.2)
Gly13Val (GGC > GTT)	1 (0.1)	1 (0.1)	2 (0.1)
Gly13Arg (GGC > CGC)	1 (0.1)	2 (0.2)	3 (0.1)
Gly12Ser (GGT > TTT)*	-	-	1

*Missing data.

### Association between gender, *KRAS* mutation and age

Our results have shown that the Gly12Asp mutation was the most prevalent for males and females, thus we further investigated this by analysing the overall incidence of mutant cases according to gender. The most striking result observed in our database was the fact that *KRAS* mutations affected more women compared to men (34.8% vs. 32.5%, respectively, p = 0.03) as can be seen in Table 4.

**Table 4:** ***KRAS *** **status according to gender**

**Gender**	***KRAS *** **wild-type n (%)**	***KRAS *** **mutant n (%)**	**Total n (%)**
	**5,174 (66.4)**	**2,623 (33.6)**	**7,797 (100)**
Female	2,440 (65.2)	1,305 (34.8)	3,745 (100)
Male	2,734 (67.5)	1,318 (32.5)	4,052 (100)

This result led us to stratify the mutant cases also according to age, since sex hormones play an important role during a life cycle especially during female reproductive events. The age groups were ≤40, 40–50, 50–60, 60–70 and ≥70.

**Figure 2:**
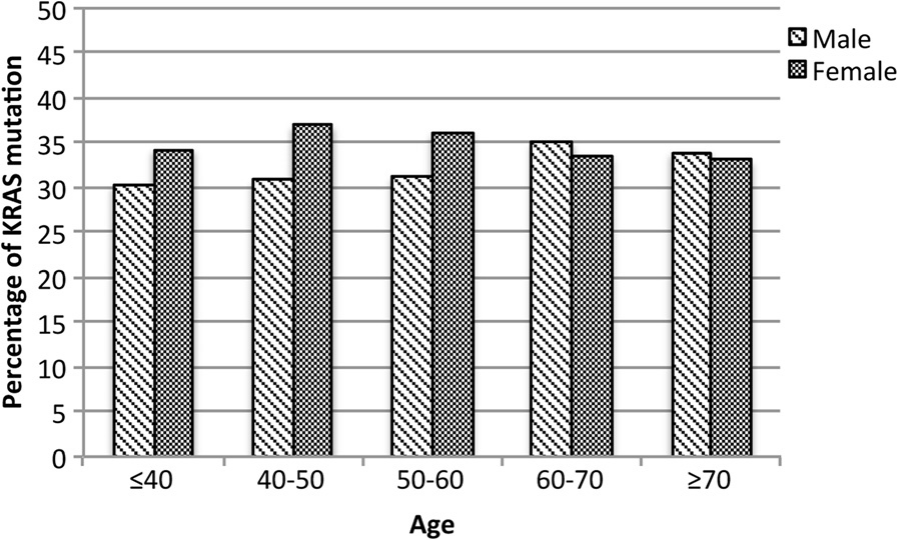
**Percentage of mutant *KRAS *cases according to sex and age.** Variables such as gender and age were analysed and the age ranges for both sexes were: ≤40 (p = 0.27), 40–50 (p = 0.02), 50–60 (p = 0.02), 60–70 (p = 0.47) and ≥70 (p = 0.73).

Figure 2 examines the mutational status differences observed according to age and sex. Corroborating results shown in Table 4, the percentages of mutations in females are higher for the ages ≤40 (p = 0.27), 40–50 (p = 0.02) and 50–60 (p = 0.02) when compared to males. In contrast, ages 60–70 (p = 0.47) and ≥70 (p = 0.73) showed a different result, where more males have mutations. However, in this case the differences in percentage within the age ranges were less dramatic (60–70 = 35.1% *vs* 33.6%; ≥70 = 33.9% *vs* 33.1%; male *vs* female) than the ones observed between males and females in the range ≤40 (30.3% *vs* 34.3%; p = 0.27), 40–50 (30.8% *vs* 37%; p = 0.02), 50–60 (31.3% *vs* 36.1%; p = 0.02).

## Discussion

In the colorectal carcinogenesis model initially described by Vogelstein [20], specific genetic events would be related to morphological tissue changes. Among these genetic changes, different studies have shown that mutations in the *KRAS* gene were found in over 30% of CRC and advanced adenomas [21,22]. The present study is consistent with this showing that among the 8,234 cases analysed, a total of 2,623 (31.9%) corresponded to mutant *KRAS* (Figure 1). From the 2,623 mutated cases, 83% were in codon 12 versus 17% in codon 13, being the aminoacid change Gly to Asp the most common for both codons and genders (Table 3). The prevalence of Gly12Asp mutation over other mutations corroborates with data from other populations around the world [23,24]. Despite variations in the frequency of types of mutations per region (Table 2), our results showed that more than 30% of the patients in each region harboured *KRAS* mutations (Table 1). Regarding prognosis, previously published data showed that Gly12Val substitutions are more aggressive having a poorer prognosis than those with Gly12Asp mutation, thus revealing a connection between survival and *KRAS* mutation type [23,25-27]. In relation to codon 13, some studies have shown that mutations in this codon could be less aggressive than in codon 12 and that patients with *KRAS* Gly13Asp mutant tumours could benefit from anti-EGFR therapies [28,29]. In addition, recent in vitro studies have confirmed that Gly13Asp mutations are associated with sensitivity to anti-EGFR antibody treatments [30,31]. Unfortunately, we were unable to provide prognosis comparisons since we could not retrieve the follow up from many patients in our cohort.

Gender was another variable we analysed in our cohort. The percentages of *KRAS* mutations pointed out a female over male prevalence (Table 4, p < 0.05). Actually, the female predominance of *KRAS* mutations in CRC patients has been reported in few recent studies performed in smaller cohorts from Asia. In China, a cohort of 167 CRC patients were tested for mutations on *KRAS* codons 12 and 13 and their results detected a higher rate of *KRAS* mutations in female compared to male patients [32] and this finding did not significantly correlate with the patient age, tumour site, differentiation grades and histological types. Differently to our data, their results referred to Gly13Asp as the most frequently mutation identified [32]. In another Chinese study, Shen et al. [33] observed that in a cohort of 118 CRC patients, there was also a higher incidence of *KRAS* mutations in female patients compared to male patients (44.7% *vs* 28.2%, p = 0.037). In Japan, researchers suggested that gender and age were independent risk factors for *KRAS* mutations [34]. Another publication did not show correlation between gender and *KRAS* mutational status [35], and one possible explanation for this could be the ethnological differences in populations studied. Nevertheless, it is important to address that the greatest prevalence of *KRAS* mutation in women has been previously observed in other cancer types such as in patients with lung adenocarcinoma, leading to the hypothesis of possible hormonal influence [36].

Clinical evidence suggests potential sex-related differences in the development and prognosis of CRC, which could be associated with sex hormones. Estrogens and androgens regulate growth, differentiation and functioning of different tissues, including the gastrointestinal tract. Estrogen is an important mitogen capable of sending its signal to the nucleus via interaction with estrogen receptors (ER) on target cells. The two distinct estrogen receptors (ERα and ERβ) tend to respond differently to estrogen. The high proliferative activity triggered by estrogen is related to its connection to the ERα, which can help tumoral development by increasing the probability of genetic mutations [37]. In contrast, the ERβ forms heterodimers with the ERα blocking their proliferative activity by suppression of oncogenic transcription factors (e.g. c-myc, cyclin D1 e cyclin A) and by stimulating the expression of tumour suppressing genes (e.g. p21 e p27) [38]. The expression of the ERβ is significantly reduced in adenomatous tissues and in colon tumours, when compared to normal mucosa in both genders, however with a slight reduction in females (p < 0.002) [39-41]. Nevertheless, there are no observed changes in the expression of ERα between the different tissues. Also in pre-cancerous lesions with a high risk of CRC development, a decrease in ERβ expression could indicate a promoting factor for the development of cancer [42]. In hereditary nonpolyposis colon cancer syndrome (HNPCC) characterised by a dominant susceptibility acquired in the early appearance of symptoms of CRC, the average age for CRC diagnosis is earlier in males than females (38.8 *vs* 47.2; p < 0.05) [19], indicating that female sexual hormones could be acting as protective factors. This estrogen protectiveness was also the conclusion after guinea pigs that were treated with PhiP (2-Amino-1-methyl-6-phenylimidazo [4,5-b] pyridine), an inducing agent for colon cancer, resulted in the average number of aberrant crypt foci higher in males than in females (p < 0.001) [43]. In regard to hormone replacement therapy, meta-analysis studies have confirmed the reverse association with the risk of developing CRC [44-47].

The role that male sexual hormones play in the risk for CRC is still unclear. There is evidence of reverse association between the serum level of the dehydroepiandrosterone sulfate and the risk of CRC [48]. The increase of CAG trinucleotide repetitions in the coding sequence of the androgen receptor seems to be related to the lowest trancriptional activation of this receptor resulting in a lower androgenic action on the tissues, increasing the risk of developing colon cancer [49]. Gillessen et al. [50] confirmed in a large retrospective evaluation of 107,859 patients with prostate cancer that patients treated with Gonadotrophin-releasing hormone (GnRH) antagonist or orchiectomy presented an increase of 30-40% risk of developing CRC when compared to the subgroup that were not submitted to androgenic deprival.

Kato et al. [51,52] showed that the estrogen receptor is involved in the *KRAS* mediated transcription and its implication in the senescence escape. Furthermore, an association between *KRAS* and estrogen receptor was also observed when mutant *KRAS* (Gly12Val) was overexpressed in NIH-3 T3 cells, which resulted in increased levels of the endogenous estrogen receptor. In addition, the *RAS* signal intensified the estrogen receptor activity as a transcription factor leading to cell transformation [53]. In a pre-clinical study it was shown that RAS oncogenes might remain latent in the mammary gland of guinea pigs until estrogen exposure. This suggests that normal proliferative processes, such as the ones induced by estrogen in the development of the mammary gland, could be necessary to lead cells with the latent RAS oncogenes to neoplastic development [54]. Collectively, these findings could indicate an association between mutant *KRAS* and sexual hormones.

Our observations of differences in *KRAS* mutational status according to sex led us to analyse also age. *KRAS* was more frequently found mutated in females than males for the ages ≤40, 40–50, and 50–60 (Figure 2). The age range 40–60 coincides with the menopausal period, which is associated with a drop in estrogen levels. In contrast, ages 60–70 and ≥70 showed a different result, where males had more mutations than females (p > 0.05). Although the p value was higher for the ages 60–70 and ≥70, this result could indicate an age-linked difference associated with several causes including men andropause. Actually, a recent study suggested that men with lower androgenicity (resulting either from reduced androgen receptor activity or lower circulating dehydroepiandrosterone sulfate) have a higher risk for colorectal cancer, however they did not correlate the hormonal influence with levels of gene mutations [55]. One theory to explain our results could be that male and female hormones may act as protective factors not exerting pressure on the expansion of *KRAS* mutant cells. However, upon menopause and andropause (events that occur at different ages for males and females) the decrease in hormonal levels could generate a pressure to stimulate a molecular switch in favour of clonal selection of cells containing *KRAS* mutations. Nevertheless, future studies on a large cohort corresponding to late age ranges are necessary to further confirm these results.

## Conclusions

We found that variables such as region, age and sex-linked factors can correlate with *KRAS* mutational status. This is the largest study to point out a statistically difference in the prevalence of *KRAS* mutations between genders in a cohort of brazilian mCRC patients. The exact interaction between *KRAS* mutations, sexual hormones and the development of CRC is still not well defined. In the adenoma-carcinoma sequence, the proliferative hormonal exposure in the presence of ERα and the decrease of ERβ could be a factor to select *KRAS* mutant clones in the adenocarcinoma histologic subtype in females. Although estrogen could serve as a protective hormone in CRC, it might not prevent mutations in *KRAS*. Our group is currently evaluating experimentally the reasons behind the differences observed in this study. Hopefully, future research will be able to elucitate the molecular links between hormones, *KRAS* mutations, age and CRC development.

## Abbreviations

CRC: Colorectal cancer; ER: Estrogen receptors; GnRH: Gonadotrophin-releasing hormone; PhiP: 2-Amino-1-methyl-6-phenylimidazo [4,5-b] pyridine.

## Competing interests

We acknowledge that there are no known conflicts of interest associated with this publication.

## Authors’ contributions

Study design and coordination: CGF, MZ. Acquisition of data: CGF, VA, IZR, APV, JHS, MHB, FMV, MZ. Statistical analysis: JHS. Analysis and interpretation of data: CGF, VA, FMV. Wrote the paper: VA, FMV, CGF, MZ. Critical revision of manuscript: CGF, VA, IZR, APV, JHS, MHB, FMV, MZ. Final approval of manuscript: CGF, VA, IZR, APV, JHS, MHB, FMV, MZ. All authors read and approved the final manuscript.

## Pre-publication history

The pre-publication history for this paper can be accessed here:

http://www.biomedcentral.com/1471-230X/14/73/prepub
